# Pathophysiological Alterations of Redox Signaling and Endocannabinoid System in Granulocytes and Plasma of Psoriatic Patients

**DOI:** 10.3390/cells7100159

**Published:** 2018-10-06

**Authors:** Ewa Ambrożewicz, Piotr Wójcik, Adam Wroński, Wojciech Łuczaj, Anna Jastrząb, Neven Žarković, Elżbieta Skrzydlewska

**Affiliations:** 1 Department of Analytical Chemistry, Medical University of Bialystok, 15-222 Bialystok, Poland; ewa.ambrozewicz@umb.edu.pl (E.A.); piotr.wojcik@umb.edu.pl (P.W.); wojciech.luczaj@umb.edu.pl (W.Ł.); anna.jastrzab@umb.edu.pl (A.J.); 2 Dermatological Specialized Center "DERMAL" NZOZ in Bialystok, 15-453 Bialystok, Poland; adam.wronski@dermal.pl; 3 LabOS, Rudjer Boskovic Institute, Laboratory for Oxidative Stress, 10000 Zagreb, Croatia

**Keywords:** psoriasis, arthritis, inflammation, granulocytes, redox signaling, oxidative stress, lipid peroxidation, 4-hydroxynonenal, lipids, endocannabinoid system

## Abstract

Inflammatory granulocytes are characterized by an oxidative burst, which may promote oxidative stress and lipid modification both in affected tissues and on a systemic level. On the other hand, redox signaling involving lipid peroxidation products acting as second messengers of free radicals play important yet not fully understood roles in the pathophysiology of inflammation and various stress-associated disorders. Therefore, the aim of this study was to evaluate the onset of oxidative stress and alterations of enzyme-dependent lipid metabolism resulting from redox imbalance in granulocytes and plasma obtained from patients with psoriasis vulgaris or psoriatic arthritis in comparison to the healthy subjects. The results obtained revealed enhanced activity of pro-oxidant enzymes nicotinamide adenine dinucleotide phosphate (NADPH) and xanthine oxidases in granulocytes with a decrease of enzymatic and non-enzymatic antioxidants in the plasma of psoriatic patients. The nuclear factor erythroid 2–related factor 2 (Nrf2) and its regulators were increased in both forms of psoriasis while heme oxygenase 1 levels were increased only in psoriasis vulgaris. The redox imbalance was associated with decreased levels of phospholipids and of free polyunsaturated fatty acids but with enhanced activity of enzymes involved in lipid metabolism (phospholipase A2, acetylhydrolase PAF, cyclooxygenases 1 and 2) and increased lipid peroxidation products 4-hydroxynonenal, isoprostanes, and neuroprostanes. Increased endocannabinoids and G protein-coupled receptor 55 were observed in both forms of the disease while expression of the cannabinoid type 1 receptor (CB1) was increased only in patients with psoriatic arthritis, which is opposite to the cannabinoid type 2 receptor. This receptor was increased only in psoriasis vulgaris. Changes in protein expression promoted the apoptosis of granulocytes by increased caspases mainly in psoriasis vulgaris. This study indicates that inhibition of the Nrf2 pathway in psoriatic arthritis promotes a redox imbalance. In addition, increased expression of CB1 receptors leads to increased oxidative stress, lipid modifications, and inflammation, which, in turn, may promote the progression of psoriasis into the advanced, arthritic form of the disease.

## 1. Introduction

Under physiological conditions, cells are in a stable state of redox homeostasis, which is maintained by the balance between continuous production of reactive oxygen species (ROS) and antioxidant activities [1]. Interactions between ROS and antioxidants produce metabolic responses to endogenous and/or exogenous signals/stressors. These signals trigger and modulate the appropriate adaptation processes or activation of the mechanisms causing cellular decay [2]. Therefore, cellular redox homeostasis plays a key role in physiology of the cell as well as in numerous pathophysiological processes. Elevated ROS levels that cannot be counteracted by the cellular antioxidant abilities induce a redox imbalance that leads to oxidative stress [1,3], which further causes oxidative modifications in the structure and function of cellular components especially of the unsaturated bio-membrane lipids [4]. The consequence is an increase of the products of ROS-dependent oxidative fragmentation as well as cyclization of fatty acids [5,6]. Among them, reactive aldehydes, which are products of oxidative fragmentation, may act as second messengers of free radicals and regulatory signaling molecules that modify cellular metabolism and may cause death either through necrosis or apoptosis [7]. Additionally, lipids can also undergo enzymatic transformation involving enzymes like phospholipases, cyclooxygenases, or lipoxygenases, which generates a large group of lipid mediators [8]. This may show pro-inflammatory or anti-inflammatory properties. Among these, endocannabinoids and leukotriene B4 (LTB4) are known as pathophysiological activators of granulocytes [9]. Endocannabinoids are agonists of G protein-dependent cannabinoid receptors such as CB1 or CB2 and receptors belonging to the TRPVs and GRPs groups whose activation modifies the level of ROS and pro-inflammatory mediators [10,11]. Activation of CB1 receptors stimulates the generation of ROS and TNF-α while CB2 and TRPV1 receptors have anti-inflammatory activity and contribute to the reduction of ROS levels [10,11]. Additionally, one of the main endocannabinoids, 2-AG, is directly able to impair both inflammation as well as oxidative stress, which is often considered to be a compensatory or adaptive mechanism [12]. Thus, changes in the endocannabinoid system may model oxidative stress and the inflammatory processes. This is supported by the fact that, among inflammatory diseases, those accompanied by acute or chronic oxidative stress exhibit higher levels of endocannabinoids [13]. Accordingly, it was suggested that endocannabinoids play a role in the pathogenesis of obesity, atherosclerosis, and diabetes, which are all comorbidities associated with psoriasis.

Psoriasis is a chronic, inflammatory disease manifested by the presence of skin lesions associated with hyper-proliferation and impaired apoptosis of keratinocytes. The disease is not only manifested by skin changes but also by systemic abnormalities like enhanced activation of leukocytes and higher levels of cytokines [14,15]. Moreover, there is a significant increase in granulocyte levels in the blood of patients with psoriasis [16]. Activated granulocytes especially neutrophils are characterized by the overproduction of ROS, which is denoted as an oxidative burst. Therefore, in many cases, their enhanced activation in the course of inflammation correlates with the intensity of oxidative stress, which is an important pathophysiological factor of inflammatory, stress-associated diseases [17,18,19,20,21] while alterations of antioxidants are associated with psoriasis [15]. In addition, it is suggested that oxidative stress may activate mechanisms that restore the redox balance including activation of the Nrf2 transcription factor, which is responsible for antioxidant and cyto-protective gene transcription [22,23]. However, a reduction in Nrf2 activity was observed in the skin of psoriatic patients [24].

Psoriasis occurs most often in two forms: as psoriasis vulgaris and as psoriatic arthritis with different clinical courses. At the same time, biochemical and metabolic differences between the two forms are not well investigated [15]. So far, there are no studies assessing possible interrelations between oxidative stress parameters, inflammatory processes, and the endocannabinoid system in patients with psoriasis. Therefore, the aim of this study was to compare the relationship between the redox balance and the endocannabinoid system with pro-inflammatory factors in granulocytes and the plasma of healthy people and the plasma of patients with psoriasis. In addition, the molecular mechanisms of the development of two different forms of psoriasis vulgaris (Ps) and psoriatic arthritis (PsA) will be compared.

## 2. Materials and Methods

The blood samples were collected from a group of 68 patients (32 women and 36 men) with a mean age of 38.2 years (range 17–66 years of age) with a diagnosis of psoriasis vulgaris for at least 6 months with at least 10% of the total body surface area affected. The other group was comprised of 34 patients (15 women and 19 men) and their mean age was 37.7 years (range 14–67 years of age) with a diagnosis of psoriatic arthritis. The control group consisted of 34 healthy subjects (15 women and 19 men) with an average age of 37.9 years old (range 20–64 years of age). None of the patients or healthy subjects had received topical or oral medications during the 4 weeks before the study. While taking the history of disease of each patient, particular attention was given to current use of certain medications (anticoagulants and antiplatelet drugs) and comorbidities (liver, kidneys, or cardiovascular diseases, cancer, respiratory disorders, and diabetes). Hence, individuals whose case history indicated any chronic or acute disorder that might affect the analyses were excluded from the study. None of the participants were smokers. The study was approved by the Local Bioethics Committee in the Medical University of Bialystok (Poland), No. R-I-002/289/2017. Written informed consent was obtained from all the patients.

Blood samples were taken into ethylenediaminetetraacetic acid (EDTA) tubes and a two-stage centrifugation was carried out. In the first stage, the sample was centrifuged at 3000× *g* (4 °C) to obtain the plasma and the buffy coat. Granulocytes were isolated from the buffy coat by gradient centrifugation using Gradisol G (Aqua-Med ZPAM–KOLASA, Łódź, Poland) Samples were layered on Gradisol and subjected to 25 min centrifugation at 300× *g* at room temperature. The individual cell fraction was collected, washed, and re-suspended in PBS containing a proteasome inhibitor mix. An antioxidant-butylhydroxytoluene (BHT) was added to plasma and granulocyte samples before storing them to prevent oxidation. Samples were stored at –80 °C until analysis.

### 2.1. Pro-Oxidant Parameters

NADPH oxidase (NOX—EC 1.6.3.1) activity was measured in granulocytes using the lucigenin-enhanced chemiluminescent method. Enzyme specific activity was expressed in RLU (Relative Luminescence Units) per microgram of proteins [25]. 

Xanthine oxidase (XO—EC 1.17.3.2) activity was determined in granulocytes as the rate of uric acid generation from xanthine detecting at a wavelength of 292 nm [26]. One unit of XO activity was defined as 1 µmol uric acid produced per min at 37 °C. Obtained data were normalized to 1 milligram of protein.

### 2.2. Antioxidant Parameters

#### 2.2.1. Determination of Protein Antioxidants 

Plasma glutathione peroxidase (GSH-Px—EC.1.11.1.6) activity was measured spectrophotometrically using the method of Paglia and Valentine [27]. GSH-Px activity was assayed by measuring the conversion of NADPH to NADP^+^. One unit of GSH-Px activity was defined as the amount of enzyme catalyzing the oxidation of 1 µmol NADPH per min at 25 °C and a pH of 7.4. Enzyme specific activity was expressed in units per mg of protein.

Plasma glutathione reductase (GSSG-R—EC.1.6.4.2) activity was measured spectrophotometrically, according to the method of Mize and Longdon [28] by monitoring the oxidation of NADPH at 340 nm at pH 7.4. One unit of GSSG-R oxidized 1 µmol of NADPH/min at 25 °C and pH 7.4. Enzyme activity was expressed in units per mg of protein.

Plasma superoxide dismutase (Cu/Zn–SOD—EC.1.15.1.1) activity was determined spectrophotometrically, according to the method of Misra and Fridovich [29]. One unit of SOD was defined as the amount of the enzyme, which inhibits epinephrine oxidation to adrenochrome by 50%. Enzyme specific activity was expressed in units per mg of protein.

Thioredoxin reductase (TrxR—EC.1.8.1.9) activity in granulocytes was estimated using a commercial assay kit (Sigma-Aldrich, St. Louis, MO, USA). The assay was based on reduction of 5,5′-dithiobis(2-nitrobenzoic) acid by NADPH to 5-thio-2-nitrobenzoic acid, which was estimated by a colorimetric measurement at 412 nm [30]. Obtained data were normalized to 1 milligram of protein.

The Thioredoxin (Trx) level in granulocytes was quantified by using the ELISA method [31]. ELISA plates with samples were incubated overnight with a primary antibody against thioredoxin (Abcam, Cambridge, MA, USA) and for 1 h with secondary goat anti-rabbit antibody (Dako, Carpinteria, CA, USA). As a chromogen, 0.1 mg/mL TMB in citric buffer with 0.012% H_2_O_2_ was added. The reaction was stopped by sulfuric acid and absorption was read at 450 nm. The Trx level was normalized for milligrams of protein. 

#### 2.2.2. Nrf2 Pathway Parameters

The expression of Nrf2 pathway parameters such as Nrf2, Keap1, Bach1, KAP1, p62, p21, and HO-1 was estimated using Western blot analysis (details are given below).

#### 2.2.3. Determination of Low Molecular Antioxidants

Reduced GSH content in plasma was measured according to the procedure of Maeso by using capillary electrophoresis [32]. The separation was performed on a fused-silica capillary (75 µm (i.d.) × 30 cm (total length)/10 cm (length to detector)) with a spectrophotometer detection at 200 nm.

The vitamins C [10] as well as vitamins A and E [11] in the plasma were determined by the HPLC methods with spectrophotometric detection at 250 nm and 294 nm, respectively.

### 2.3. Phospholipid Metabolism 

#### 2.3.1. Lipidomic Analysis of Phospholipids 

Eight plasma samples of patients with *psoriasis vulgaris*, eight patients with psoriasis arthritis, and eight plasma samples from a control group were used to estimate phospholipid profiles of each group. Total lipids from all plasma samples were extracted by a modified Folch method [33]. The total amount of phospholipid (PL) was quantified with a phosphorus assay and performed according to Bartlett and Lewis [34]. The phospholipid profile was characterized by Hydrophilic interaction liquid chromatography (HILIC-LC)-MS performed on an UPLC system (Agilent 1290, Santa Clara, CA, USA) coupled to a quadrupole time of flight mass spectrometer (Agilent, QTOF 6540). We have described this method in details previously [35]. Phospholipid molecular species were identified according to the typical fragmentation pathways [36]. The relative abundance of each ion was calculated by normalizing the area of each extracted ion chromatogram peak to the area of an internal standard.

#### 2.3.2. Phospholipid Profile

We analyzed the results obtained for a plasma phospholipid profile of both groups of psoriatic patients versus healthy volunteers using a Partial least squares-discriminate analysis (PLS-DA) and variable importance in projection (VIP) for the estimation of the importance among each variable, which were driving the separation of examined groups (MetaboAnalyst version 3.0) [37].

#### 2.3.3. Determination of Phospholipid and Free Fatty Acids Profile

Fatty acids were determined as fatty acid methyl esters by gas chromatography using an FID detector after lipid fraction isolation by Folch extraction and a thin layer chromatography technique separation of free fatty acids (FFA) and phospholipids (PL) [38]. Quantitation was achieved by using an internal standard method (ISTD).

#### 2.3.4. Determination of Enzymes Metabolizing Phospholipids

Phospholipase A2 (PLA2—EC.3.1.1.4) activity was measured spectrophotometrically by using a PLA2 Assay Kit (No. 765021, Cayman Chemical Company, Ann Arbor, MI, USA), according to kit instructions [39].

PAF acetylhydrolase (PAF-AH—EC.3.1.1.47) activity was measured spectrophotometrically by using the Cayman's PAF Acetylhydrolase Assay Kit (No. 760901, Cayman Chemical Company, Ann Arbor, MI, USA), according to kit instructions [40].

Cyclooxygenase 1 and 2 (COX-1/2—EC.1.14.99.1) activities were measured spectrophotometrically by using a commercial assay kit (Cayman Chemical Company, Ann Arbor, MI, USA) [41]. For distinguishing COX-1 activity from COX-2 activity, the specific COX-1 inhibitor SC-560 was used [42].

### 2.4. Lipid Peroxidation

Products of phospholipid fragmentation (low molecular aldehydes) were measured by GC/MS in plasma using the selected ion monitoring (SIM) mode while the *O*-PFB-oxime or *O*-PFB-oxime-TMS derivatives were measured by using minor modifications of the method of Luo et al. [43]. The used ions were as follows: *m*/*z* 333.0 and 181.0 for 4-HNE-PFB-TMS, *m*/*z* 204.0.

The determination of products of phospholipid cyclization (total F_2_-isoprostanes (8-isoPGF_2α_) and A_4_/J_4_-neuroprostanes (NPs)) were based on methods of Coolen and Fam [44,45]. 8-isoPGF_2α_ was analyzed in a negative-ion mode using the MRM mode: *m*/*z* 353.2→193.1 (for 8-isoPGF_2α_) and 357.2→197.1 (for 8-isoPGF_2 α_-d_4_) while NPs used the ion monitoring (SIM) mode in the *m*/*z* 357.0 as a series of peaks.

### 2.5. Estimation of the Endocannabinoid System

The LC-MS/MS method was used for the quantification of the levels of endocannabinoids in plasma [46]. Endocannabinoids were analyzed in positive-ion mode using the MRM mode (Shimadzu LCMS 8060, Shimadzu, Kioto, Japan). Transitions of the precursor to the product ion was as follows: *m*/*z* 348.0→62.15 for AEA, *m*/*z* 379.0→269.35 for 2-AG, *m*/*z* 356.0→63.05 for AEA-*d*_8_, *m*/*z* 387.0→294.0, *m*/*z* for 2-AG-*d*_8_, and *m*/*z* 430.0→66.0 for OEA-*d*_4_. 

FAAH (fatty acid amide hydrolase) (EC.3.5.1.99) activity was assessed according to the procedure described by Siegmund [47] following the releasing of *m*-nitroaniline (m-NA) from decanoyl *m*-nitroaniline at 410 nm. 

MAGL (monoacylglycerol lipase) (EC.3.1.1.23) activity was assayed following the release of 5′-thio-2-nitrobenzoic acid from 340 nm [48]. 

The expression of cannabinoid receptors such as CB1, CB2, and GPR55 was estimated using Western blot analysis (details are given below).

### 2.6. Protein Modifications 

#### Determination of Protein Oxidative Modifications

Protein oxidative modifications in plasma were estimated according to the tryptophan and protein carbonyl levels. To analyze tryptophan levels, samples were diluted in 0.1 mol/L H_2_SO_4_ (1:10) and fluorescence emission/excitation at 325 nm/420 nm and 288 nm/338 nm, respectively, was measured [49]. All results were normalized for 1 milligram of protein. The levels of protein carbonyl groups were determined spectrophotometrically (370 nm) using 2,4-dinitrophenylhydrazine [49] and was expressed as nmoles of carbonyl groups per mg protein.

The amounts of 4-hydroxynonenal (4-HNE) protein adducts were measured using a genuine ELISA method and using an anti-4-HNE-His murine monoclonal antibody (anti-4-HNE-His murine monoclonal antibody, clone 4-HNE 1g4, curtesy of Prof. Georg Waeg from the University of Graz) along with a goat anti-mouse antibody (Dako, Carpinteria, CA, USA) as primary and secondary antibodies [50]. The concentrations of 4-HNE–protein adducts were normalized for 1 milligram of protein. 

### 2.7. Expression of Pro-Apoptotic and Anti-Apoptotic Proteins in the Cells

The expression of caspase-8, caspase-9, caspase-3, p53, Bcl2, cytochrome c was estimated using Western blot analysis (details are given below).

### 2.8. Pro-Inflammatory Factors 

The expression of IL-6 and IL-17 in granulocytes as well as NF-κβ and TNF-α in plasma was estimated by using Western blot analysis (details are given below).

### 2.9. Western Blot Analysis 

Western blot analysis of protein expression was performed according to Eissa and Seada [51]. The analysis was performed on samples of randomly selected healthy subjects (n = 8) and patients with psoriasis vulgaris (n = 16) and psoriatic arthritis (n = 8). IL-6 and IL-17 were determined in plasma, CB1/2 and GPR55 were estimated in granulocyte membrane fraction, and NF-κβ, TNF-α, Nrf2, Keap1, Bach1, KAP1, p62, p21, HO-1, p53, Bcl2, cyt c, caspase 3, 8, and 9 were estimated in granulocyte cytosol. β-actin and Na^+^/K^+^-ATPase (for cytosolic and membrane fractions, respectively) were used as internal loading controls. Samples were electrophoretically separated on 10% gels, transferred to 0.2 µm pore-sized nitrocellulose, and incubated overnight with primary antibodies against: Bach1, HO-1, caspase-9, p21, KAP1, GPR55, IL-17 (host: rabbit) and NF-κβ, TNF-α, p62, caspase-3, IL-6, β-actin, and Na^+^/K^+^-ATPase (host: mouse) that were purchased from Sigma-Aldrich, (St. Louis, MO, USA). Primary antibodies against: Keap1 (host: goat), cyt-c, Bcl2, p53, caspase-8 (host mouse), CB1, CB2, and Nrf2 were purchased from Santa Cruz Biotechnology (Santa Cruz, CA, USA). Next, membranes were incubated for 2 h with alkaline phosphatase secondary IgG antibody against a corresponding primary antibody (Sigma-Aldrich, St. Louis, MO, USA). Protein bands were visualized using the BCIP/NBT Liquid substrate system (Sigma-Aldrich, St. Louis, MO, USA), which was determined using the VersaDoc System and Quantity One software (Bio-Rad Laboratories Inc., Hercules, CA, USA). The results are expressed as a percentage of the expression determined in control cells.

### 2.10. Statistical Analysis

Data obtained in the current study were expressed as mean ± SD. For comparisons between groups, the chi-square test was used for categorical variables. The normal distribution of quantitative data was verified by using the Kolmogorov-Smirnov test with corrections performed using the Lilliefors test and the Shapiro-Wilk test. To compare differences between the groups, the Mann-Whitney *U* test and Kruskal-Wallis test were used. For the comparison of dependent variables, Friedman’s test was used with an adjusted Conover post-hoc test. A *p*-value of <0.05 was considered statistically significant. All statistical analyses were performed by using Stata/IC 13.0 (StataCorp, College Station, TX, USA).

## 3. Results

### 3.1. Redox Balance

The onset of psoriasis promoted the formation of oxidative stress both in plasma and in granulocytes of patients with both forms of psoriasis: psoriasis vulgaris (Ps) and psoriatic arthritis (PsA) (Table 1). Namely, both forms of psoriasis were associated with a significant increase in granulocyte XO and NADPH oxidase activity and with a decrease of the activity/level of protein antioxidants (TrxR and Trx) and of low molecular antioxidants (GSH, vitamin C, and vitamin E) in plasma. Vitamin A levels were significantly decreased only in PsA and GSH-Px activity was decreased only in Ps patients while Cu,Zn-SOD activity was significantly higher in the plasma of Ps patients. 

**Figure 1:**
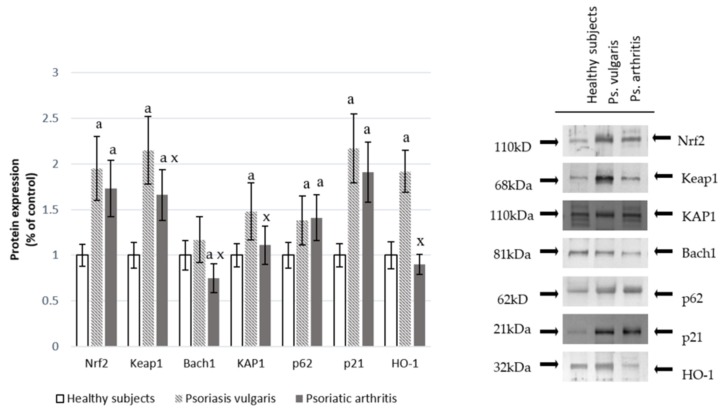
The level of the Nrf2 pathway factors in granulocytes from healthy subjects (n = 8) and psoriatic patients (psoriasis vulgaris (n = 16) or psoriatic arthritis (n = 8)). ^a^ *p* < 0.05 when compared with healthy subjects. *^x^ p* < 0.05 when compared with patients with psoriasis vulgaris.

Changes at the protein antioxidant levels were also accompanied by changes in the expression of the transcription factor Nrf2 in granulocytes, which is responsible for the transcription of anti-oxidative proteins. This was shown in Figure 1. Significant increases in Nrf2 and its target protein HO-1 in granulocytes of Ps and an increase in Nrf2 accompanied by a decrease in HO-1 in granulocytes of PsA patients were observed. The level of the granulocyte cytosolic Nrf2 inhibitor, Keap1, was increased in both Ps and PsA while the nuclear inhibitor, Bach1, was reduced in PsA patients. However, the Nrf2 activators KAP1, p21, and p62 were enhanced in both types of psoriasis.

### 3.2. Phospholipid Metabolism 

**Figure 2:**
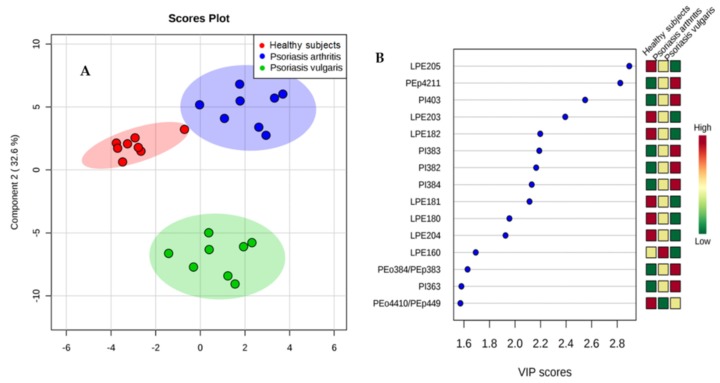
(**A**) Partial least squares-discriminate analysis (PLS-DA) plot of the phospholipid species and relative abundance determined by HILIC-LC-MS in the plasma of healthy people and both groups of psoriatic patients. The red triangles indicate healthy subjects (n = 34) while the green and blue crosses represent patients with psoriasis vulgaris (n = 68) and psoriatic arthritis (n = 34), respectively. (**B**) Graphical presentation of the regulation of phospholipids with VIP score ≥1, which differentiate healthy subjects and patients with psoriasis vulgaris and psoriatic arthritis.

The PLS-DA plot showed a good graphical separation of the major phospholipid species in healthy subjects and both groups of psoriatic patients, which allows for an evident distinction between them (Figure 2A). Fifteen phospholipid species with VIP ≥1 concerning species, particularly, from lysophosphatidylethanolamine (LPE) and phosphatidylinositol (PI) were downregulated or upregulated in the plasma of psoriatic patients, respectively (Figure 2B).

### 3.3. Fatty Acids Profile

Changes in phospholipid profiles observed in plasma of Ps and PsA patients were associated with changes in fatty acids composition (Table 2). Phospholipid LA (18:2) and LA (18:3) as well as free AA (20:4) and DHA (22:6) levels were significantly decreased in Ps and PsA compared with healthy subjects and, additionally, these fatty acid levels were significantly reduced in PsA compared to Ps. Furthermore, phospholipid AA (20:4) and DHA (22:6) levels were significantly lowered in PsA if compared with a healthy control. 

Activities of all enzymes participating in phospholipid metabolism (PLA2, PAH-AH, COX-1, COX-2) were significantly increased in both diseases and additionally were significantly higher in the plasma of Ps than PsA patients. 

### 3.4. Lipid Peroxidation

Changes in PUFA levels were accompanied by enhanced levels of lipid oxidative fragmentation products such as 4-HNE as well as products of phospholipid oxidative cyclization such as 8-isoprostanes and neuroprostanes observed in plasma of Ps and PsA patients but only low molecular aldehyde levels were significantly higher in Ps than in PsA patients (Table 3). Moreover 4-HNE-protein adduct levels were significantly increased in the plasma of patients suffering from both types of disease but were higher in Ps than PsA patients.

### 3.5. Endocannabinoid System

**Figure 3:**
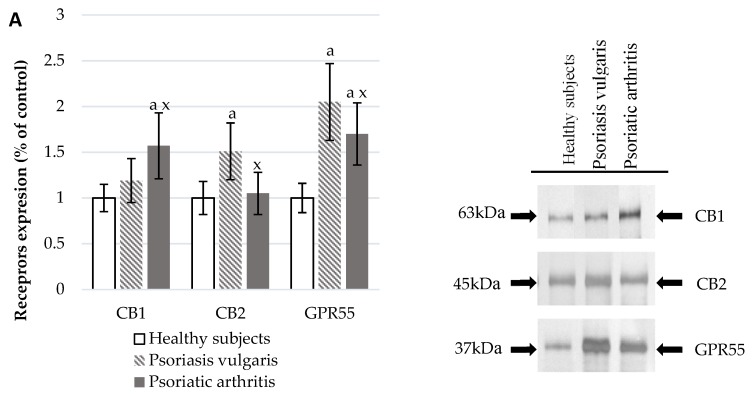

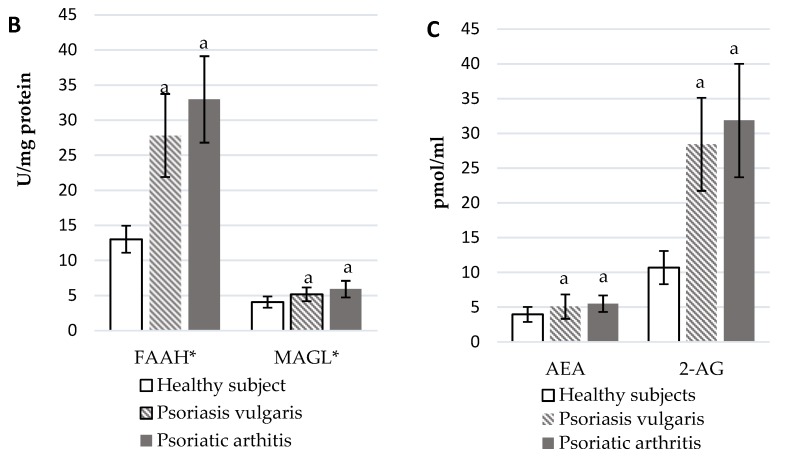
Endocannabinoid system receptor expression in granulocytes* of healthy subjects (n = 8) and patients with psoriasis vulgaris (n = 16) and psoriatic arthritis (n = 8) (**A**), endocannabinoid levels, (**C**) and activity of enzymes metabolizing endocannabinoids in plasma (**B**) of healthy subjects (n = 34) and patients with psoriasis vulgaris (n = 68) and psoriatic arthritis (n = 34). ^a^ *p* < 0.05 when compared with healthy subjects and ^x^ *p* < 0.05 when compared with patients with psoriasis.

The development of Ps and PsA also affected the functioning of the endocannabinoid system (Figure 3). In the plasma of Ps and PsA patients, there were significantly higher levels of AEA and 2-AG than in healthy subjects (Figure 3C). The activity of granulocyte enzymes degrading endocannabinoids including FAAH and MAGL was also increased in patients (Figure 3B). The expression of cannabinoid receptors was also enhanced in patient’s granulocytes but CB1 was higher in PsA patients while CB2 was higher in Ps patients. The GPR55 expression was equally increased in both groups of psoriatic patients.

### 3.6. Protein Modifications

**Figure 4:**
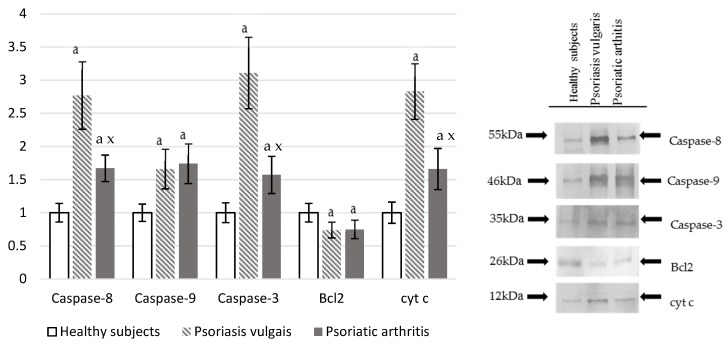
The level of pro-apoptotic proteases (caspase-8, caspase-9, caspase-3) and proteins involved in cell death (Bcl2 and cytochrome c) in granulocytes from healthy subjects (n = 8) and patients with psoriasis vulgaris (n = 16) and psoriatic arthritis (n = 8). ^a^ *p* < 0.05 when compared with healthy subjects. ^x^ *p* < 0.05 when compared with patients with psoriatic vulgaris.

Both Ps and PsA led to oxidative damage of the granulocyte cellular proteins (Figure 4), which was indicated by a significant increase in the level of protein dityrosine and enhanced caspase activities. Caspase-3, caspase-8, caspase-9, and cyt-c expression was significantly higher in psoriatic patients when compared with the control. Expression is also higher in Ps than in PsA while Bcl2 was significantly lower in Ps or PsA than in healthy control granulocytes. 

### 3.7. Pro-Inflammatory Mediators

**Figure 5:**
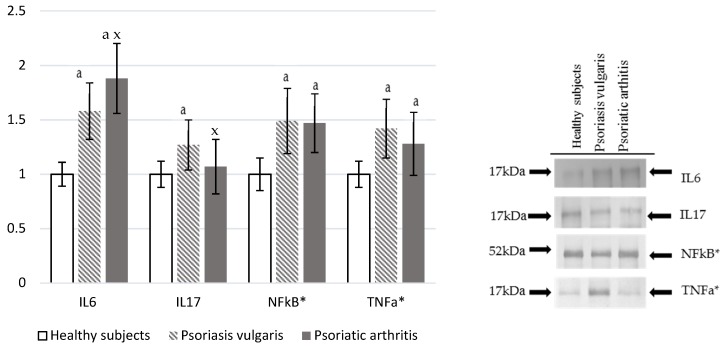
The levels of interleukins and pro-inflammatory mediators in plasma or granulocytes* from healthy subjects (n = 8) and patients with psoriasis vulgaris (n = 16) and psoriatic arthritis (n = 8). ^a^ *p* < 0.05 when compared with healthy subjects. ^x^ *p* < 0.05 when compared with patients with psoriasis vulgaris.

Ps and PsA were characterized by increased levels of pro-inflammatory markers (Figure 5). Plasma IL-6 level was significantly higher in Ps and PsA patients than in a control, which is also higher in PsA when compared to Ps levels. On the other hand, IL-17 was enhanced only in PsA patients. However, NF-κβ and TNF-α were significantly, but equally, increased in granulocytes of both Ps and PsA patients in comparison to values obtained for granulocytes of the healthy control subjects. 

## 4. Discussion

Psoriasis has a complex autoimmune pathogenesis resulting from inflammatory pathophysiology and an imbalance in the redox system homeostasis, which leads to persistent oxidative stress [15]. An important role in the pathogenesis of psoriasis is also attributed to genetic factors. It is believed that some mutations may lead to an abnormally increased activity of the immune system and, consequently, to increased inflammation and accompanying oxidative stress [15].

An important pathophysiological component of oxidative stress observed in patients with psoriasis vulgaris (Ps) and psoriatic arthritis (PsA) in our study were disturbances in phospholipid metabolism. The results of the current study indicate changes in the metabolism of PUFAs leading to disturbances in the biosynthesis of different lipid mediators including endocannabinoids generated as a result of the action of phospholipases [12] among which the activity of phospholipase A2 was found to be significantly higher in Ps then in PsA. Therefore, elevated levels of two main endocannabinoids anandamide (AEA) and 2-arachidonoylglycerol (2-AG) were observed in patients with both forms of psoriasis despite elevated activity of enzymes degrading these endocannabinoids (FAAH and MAGL). These findings suggest that higher levels of endocannabinoids are caused by their enhanced generation. Endocannabinoids, which are the agonists of specific protein G-coupled receptors, are thought to have pleiotropic effects depending on the type of the receptor activated [10]. In our patients, the expression of the receptor GPR55 was elevated in both forms of psoriasis while the expression of the receptor CB2 was elevated only in Ps in contrast to the CB1 receptor, which was increased only in PsA patients. Overexpression of endocannabinoids may be an attempt of the organism/inflammatory cells to prevent pathological conditions occurring during the progression of psoriasis. Previous in vitro studies suggested that endocannabinoids themselves may cause the inhibition of the NF-κB pathway, which may exert anti-inflammatory effects [52]. However, the level of pro-inflammatory factors and the intensity of oxidative stress were found to be so pronounced in the course of psoriasis (both forms) that the activities of endocannabinoids were unable to control such a cellular and systemic inflammatory character of oxidative stress. Consequently, an elevated expression of NF-κβ and the product of its transcription activity, TNF-α, were observed in granulocytes of both Ps and PsA. This transcription factor takes part in both inflammation and redox balance regulation. The NF-κB enhances pro-oxidative activity through the expression of NADPH oxidase, xanthine oxidase, and iNOS [53]. Our study also showed enhanced activity of NADPH and xanthine oxidases to a similar degree in both types of psoriasis, which was also the case of NF-κB. Activation of pro-oxidant enzymes revealed in granulocytes of psoriatic patients indicates that these cells undergo an oxidative burst and generates ROS in the course of both Ps and PsA. The available literature data also indicate enhanced pro-oxidative conditions in Ps [19], but there is a lack of studies focused on PsA, which was done in our study.

Since the physiological redox balance results from an equilibrium between pro-oxidants and antioxidants, the antioxidant capacity of plasma obtained from psoriatic patients has also been assessed to reflect systemic oxidative stress. Notably, the Cu,Zn-SOD activity has been shown to be increased in the plasma of both groups of psoriatic patients most likely as a response to increased superoxide anion generation by NADPH and xanthine oxidases that is metabolized by superoxide dismutases [54]. Moreover, capacities of antioxidant systems such as glutathione (glutathione peroxidase-GSH-glutathione reductase) and thioredoxin-dependent reductase (TrxR-Trx), as well as levels of supporting small molecular antioxidants including vitamins A, E, and C were found to be decreased in our patients. Earlier reports indicated a lack of changes or an increase in vitamins A and E levels [19], but these parameters are strongly influenced by eating habits and the severity of the disease. As seen in the current study, previous studies also showed lower activity of GSH-Px in plasma and granulocytes of psoriasis patients [19] and current knowledge suggests that it may result from mutations in the genes of these antioxidants [54]. However, other studies have shown that an enhanced level of NF-κβ may promote transcription of antioxidants including SOD, GSH-Px, and HO-1 [53]. This is consistent with the elevated activity of Cu,Zn-SOD and enhanced expression of Trx and HO-1 observed in the current study in patients with psoriasis vulgaris. Enhanced expression of HO-1 and other antioxidant proteins may also result from modulation of gene expression by the transcription factor Nrf2 [23]. The regulation of Nrf2 activity is related to the influence of its inhibitors and activators [22,55]. In physiological conditions, Nrf2 is coupled to the cytosolic inhibitor Keap1, which directs this transcription factor to ubiquitination and degradation and, in this way, prevents its translocation to the nucleus and initiation of the transcription process [22]. Increased Keap1 levels observed in psoriasis should mean stronger Nrf2 degradation. However, Keap1 has several critical cysteine residues that may be modified by ROS and by the lipid peroxidation product 4-HNE [56,57]. The levels of 4-HNE were revealed as increased in psoriatic patients, which might prevent Nrf2 and Keap1 binding [58]. Furthermore, the Nrf2 activators levels (KAP1, p62, and p21) were also increased in granulocytes of psoriatic patients, which indicate enhanced activation of Nrf2. Additional confirmation of the role of oxidative stress in the course of Ps are the results of clinical trials in which antioxidant supplementation resulted in a significant improvement in the general status of psoriatic patients [59]. So far, systemic changes at the transcription level have not been studied and only the local reduction of Nrf2 transcriptional activity in the skin of psoriasis patients has been found [24]. This occurred despite the markedly lower levels of Nrf2 pathway inhibitors such as Bach1 and Keap1 in PsA than in Ps patients indicating the role of activator KAP1 revealed at a significantly reduced level in granulocytes of PsA patients if compared to Ps. However, the lack of expected increase of HO-1 level in the granulocytes of patients with PsA vs. Ps suggests that, despite the increased pro-oxidative conditions, there is no enhancement of the anti-oxidative response in the inflammatory cells of these patients. In addition, HO-1 shows a direct antioxidant effect promotes more intense oxidative stress in PsA than in Ps patients.

The pro-oxidative conditions observed in both forms of psoriasis should result in ROS reactions with nucleophilic compounds including lipids and proteins, which causes their oxidative modifications. Particularly susceptible to oxidative modifications are PUFAs, which are either bound to phospholipids or as free molecules, in bio-membranes and are metabolized in ROS-dependent and in enzyme-catalyzing reactions [4]. The results obtained in our study have shown that levels of PUFAs decrease in both forms of psoriasis but more in the case of PsA, which had more pronounced onset of oxidative stress, as discussed earlier. Similar systemic alterations of the PUFA metabolism were also observed in other inflammatory diseases such as rheumatoid arthritis (RA) [60,61]. The ROS-dependent peroxidation of PUFAs generates a broad range of products including compounds produced during oxidative cyclization as well as fragmentation [6,62]. The 8-isoprostanes and neuroprostanes are generated in situ from esterified PUFAs (mainly from AA and DHA, respectively) being released by phospholipases including PLA2 and PAF-AH [63]. The activity related to this was also increased in our psoriatic patients but more in the case of Ps than PsA. The increased activity of PLA and PAF-AH may promote the release of 8-isoprostanes and neuroprostanes, which was confirmed by the significantly elevated levels of these compounds in the plasma of patients with both forms of psoriasis. That might further lead to the vicious pathophysiological circle because 8-isoprostanes act as signaling molecules activating the CD11b/CD18 and CD11c/CD18 receptors that play a key role in neutrophil activation and migration [64]. Another relevant pathway involving oxidative metabolism of PUFAs such as arachidonic acid is by cyclooxygenases. The activity of this was increased in the case of psoriasis. Since the activation of cyclooxygenases leads to the generation of pro-inflammatory eicosanoids, increased COX-1/2 activity may contribute to their over-generation in similar ways as observed for other inflammatory diseases [65].

The results obtained in this study also indicate enhanced plasma levels of other lipid peroxidation end products including reactive aldehydes generated during PUFAs fragmentation (4-HNE) with significantly higher increases in the plasma of Ps patients if compared to patients with PsA. Observed increases in the 4-HNE levels reflect non-enzymatic peroxidation of LA and AA [7] reducing their levels, which was found in the plasma of psoriatic patients. This reactive aldehyde may diffuse to longer distances than ROS and behave as a secondary messenger of free radicals that can propagate oxidative damages especially if bonded to proteins. This aldehyde reacts with the nucleophilic cysteine, lysine, and histidine residues of proteins to form stable covalent adducts [66]. Exposure of biological systems to these electrophiles can modify a subset of proteins, which generates intramolecular covalent adducts with histidine in both Ps and PsA. It is known that 4-HNE creating protein adducts could decrease biological activity of GSH-Px and GSH along with Keap1 [67]. However, it was also shown that 4-HNE, at least in vitro, reduces the oxidative burst of granulocytes interacting with proteins involved in that process [62]. Therefore, this particular aldehyde might also act in a positive way by attenuating oxidative activities of the inflammatory cells in psoriasis, which is similar to its multiple effects in cardiovascular diseases and the metabolic syndrome, which are known as psoriatic comorbidities [58,68].

Along with the actions of lipid peroxidation products as signaling molecules, free DHA may also bind to GPR120 and inhibit NF-κβ, which consequently reduces the severity of inflammation [69]. However, a decrease of free DHA has been observed for our psoriatic patients. In PsA patients, this was more pronounced when accompanied by an increase in the level of NF-κβ and inflammatory conditions, which was confirmed by an enhanced level of pro-inflammatory cytokines (Il-6, Il-17, and TNF-α). This may offer an explanation for the activity principles of the effective diets rich in DHA applied for the integrative treatment of psoriasis [70].

Protein modifications observed in psoriasis could lead to impaired functions of targeted cellular proteins, which causes apoptosis through receptor and mitochondrial pathways. Psoriatic patients showed an increase in TNF-α expression, which could promote the activation of the death receptor-mediated pathway as verified by a significant increase of the expression of caspase-8 revealed for granulocytes in both forms of psoriasis. This caspase can directly activate executioner caspase-3, but may also be indirectly involved in the mitochondrial pro-apoptotic pathway. This results in caspase-9 activation [71], which was more expressed in Ps than in PsA likely because of the strong influence of caspase-8 from the external pathway. However, caspase-8 can also activate the transcription factor, NF-κB, which is a product of transcriptional activity of TNF-α activated death receptors [72]. The enhanced expression of the executioner caspase-3 was observed in both forms of psoriasis but was significantly higher in Ps than in PsA. Moreover, granulocytes from patients with psoriasis were also characterized by changes in the mitochondrial pathway including a decrease of the anti-apoptotic protein Bcl-2 that could lead to a release of cytochrome c from mitochondria that binds APAF1 and subsequently induces caspase-9, which results in the activation of caspase-3 and, thus, executes cell death [73]. Lastly, it should be mentioned that activation of cannabinoid receptor CB2 could lead to enhanced biosynthesis of ceramides and cause overexpression of protein p38 and transcription factor 4, which consequently results in cell apoptosis [74]. This may explain a stronger expression of apoptotic pathways in inflammatory granulocytes obtained from Ps than from PsA patients.

## 5. Conclusions

This study revealed changes in the redox balance and results in modifications of phospholipid metabolism in psoriatic patients, which is associated with significant differences in several cell signaling pathways relevant for the pathophysiology of psoriasis. Oxidative stress was found as a common element of both Ps and PsA, but inflammatory cells of these patients responded to it in different ways. Granulocytes obtained from patients with Ps showed higher activation of the Nrf2 pathway and expression of CB2 that could attenuate oxidative stress and act as an anti-inflammatory process but is also indicated as more enhanced oxidative phospholipid modifications. However, more pronounced phospholipid enzymatic metabolism in PsA than in Ps patients was observed. Thus, changes in the redox system and the metabolism of granulocyte phospholipids in the course of psoriasis have a much more complex character than in other inflammatory diseases and may also be attributed to genetic factors.

## Figures and Tables

**Table 1:** The levels of oxidant and antioxidant parameters in plasma or granulocytes* from healthy subjects (n = 34) and patients with psoriasis vulgaris (n = 68) and psoriatic arthritis (n = 34).

Analyzed Parameters	Healthy Subjects	Psoriasis Vulgaris	Psoriatic Arthritis
XO* U/mg protein	18.8 ± 2.5	30.1 ± 5.3 ^a^	32.71 ± 5.73 ^a^
NADPH oxidase* U/μg protein	2.25 ± 0.28	3.21 ± 0.55 ^a^	3.92 ± 0.71 ^a^
Cu,Zn-SOD U/mg protein	3.91 ± 0.51	4.72 ± 0.81 ^a^	4.12 ± 0.53 ^x^
GSH-Px mU/mg protein	2.16 ± 0.47	1.86 ± 0.45 ^a^	2.17 ± 0.63 ^x^
GSSG-R mU/mg protein	0.73 ± 0.13	0.71 ± 0.14	0.68 ± 0.16
Trx μg/mg protein	6.45 ± 0.92	5.17 ± 0.98 ^a^	3.20 ± 0.89 ^a,x^
TrxR U/mg protein	0.97 ± 0.22	0.56 ± 0.17 ^a^	0.55 ± 0.13 ^a^
GSH nmol/mL	9.54 ± 1.24	6.35 ± 1.06 ^a^	6.19 ± 0.65 ^a^
Vitamin C nmol/mL	41.79 ± 8.69	28.22 ± 9.60 ^a^	22.82 ± 5.05 ^a,x^
Vitamin E nmol/mL	1.01 ± 0.24	0.79 ± 0.20 ^a^	0.80 ± 0.15 ^a^
Vitamin A pmol/mL	248.6 ± 22.7	228.8 ± 32.9	213.6 ± 35.2 ^a^

^a^*p* < 0.05 when compared with healthy subjects. ^x^ *p* < 0.05 when compared with patients with psoriasis vulgaris.

**Table 2:** The level of phospholipids, free fatty acids, and the activity of the enzymes that metabolize phospholipids and lipid mediators in plasma from healthy subjects (n = 34) as well as patients with psoriasis vulgaris (n = 68) and psoriatic arthritis (n = 34).

Analyzed Parameters	Healthy Subjects	Psoriasis Vulgaris	Psoriatic Arthritis
Phospholipid LA (18:2) μmol/mL	1.45 ± 0.21	1.24 ± 0.27 ^a^	1.11 ± 0.23 ^a,x^
Phospholipid LA (18:3) μmol/mL	29.95 ± 6.81	26.21 ± 7.96 ^a^	24.04 ± 7.82 ^a^
Phospholipid AA (20:4) μmol/mL	792.7 ± 124.9	739.6 ± 131.5	712.6 ± 143.9 ^a^
Phospholipid DHA (22:6) μmol/mL	292.4 ± 65.2	267.0 ± 71.4	253.5 ± 73.3 ^a^
Free LA (18:2) nmol/mL	17.46 ± 4.83	16.13 ± 5.31	15.09 ± 5.04
Free AA (20:4) nmol/mL	1.51 ± 0.32	1.32 ± 0.41 ^a^	1.09 ± 0.36 ^a,x^
Free DHA (22:6) nmol/mL	1.73 ± 0.41	1.43 ± 0.45 ^a^	1.20 ± 0.44 ^a,x^
PLA_2_ nmol/mL/min	9.18 ± 0.919	12.05 ± 1.59 ^a^	9.98 ± 1.48 ^a,x^
PAH-AH nmol/mL/min	30.27 ± 2.12	56.56 ± 10.49 ^a^	48.94 ± 7.53 ^a,x^
COX-1 nmol/mL/min	0.43 ± 0.07	0.63 ± 0.13 ^a^	0.54 ± 0.09 ^a,x^
COX-2 nmol/mL/min	0.17 ± 0.03	0.52 ± 0.14 ^a^	0.44 ± 0.09 ^a,x^

^a^*p* < 0.05 when compared with healthy subjects; ^x^ *p* < 0.05 when compared with patients with psoriasis vulgaris.

**Table 3:** The level of protein and phospholipid oxidative modification products in plasma from healthy subjects (n = 34) and patients with psoriasis vulgaris (n = 68) and psoriatic arthritis (n = 34).

Analyzed Parameters	Healthy Subjects	Psoriasis Vulgaris	Psoriatic Arthritis
4-HNE nmol/mL	8.90 ± 3.71	15.36 ± 7.81 ^a^	11.01 ± 1.86 ^a,x^
Isoprostanes pmol/mL	1.61 ± 0.37	3.41 ± 0.79 ^a^	3.84 ± 0.99 ^a^
Neuroprostanes pmol/mL	2.93 ± 0.61	6.56 ± 1.20 ^a^	6.42 ± 1.09 ^a^
Tryptophan U/mg protein	35.12 ± 5.36	26.84 ± 6.21 ^a^	24.79 ± 6.63 ^a^
HNE–protein pmol/mg protein	15.24 ± 3.62	21.59 ± 4.26 ^a^	17.45 ± 4.16 ^a,x^

^a^*p* < 0.05 when compared with healthy subjects. ^x^ *p* < 0.05 when compared with patients with psoriasis vulgaris.
